# Occupational Therapy Program With a Learning Goal That Includes Multiple Goal Categories: A Case Report of a Cancer Survivor

**DOI:** 10.7759/cureus.77847

**Published:** 2025-01-22

**Authors:** Katsuma Ikeuchi, Seiji Nishida

**Affiliations:** 1 Department of Occupational Therapy, Faculty of Health and Welfare, Prefectural University of Hiroshima, Mihara, JPN

**Keywords:** goal-setting, lymphoma, occupational therapy, palliative care, quality of life

## Abstract

Successful goal-setting in occupational therapy can enhance cancer survivors' quality of life by fostering participation in meaningful and purposeful activities. To this end, strategies to prevent goal-setting failures are essential. This report describes the case of a cancer survivor who underwent occupational therapy with the incorporation of a learning goal across multiple categories.

The participant, a 73-year-old male patient, attended four weekly one-hour occupational therapy sessions. Initially, he lacked motivation for activities beyond walking, delaying goal-setting. Solely focusing on walking-related performance goals, as derived from the participant's narrative, would likely have led to emotional decline due to low goal-achievement probability. Instead, the therapist introduced learning goals encompassing leisure, psychological, and social domains. Concretely, the participant resumed playing Goban, a cherished pastime from his youth, which fostered positive experiences and alleviated loneliness. Gradually, he re-engaged in hobbies and began participating in activities, such as karaoke and social dining, resulting in improved quality of life.

This improvement underscores the importance of setting diverse learning goals beyond a single category. Further research is needed to explore the long-term effects of this approach on goal-setting success rates and quality of life in cancer survivors with a range of cancer types.

## Introduction

Many cancer survivors have fair or poor health status, physical and psychological disabilities, and limitations in activities of daily living or instrumental activities of daily living, which may be a late consequence of cancer and its treatment [1]. As they are constrained to live with the persistent effects of cancer and its treatment, it is important to support their quality of life (QOL).

Occupational therapists are autonomous health professionals who work with individuals, groups, and communities across various settings to promote participation in occupations that provide value and meaning to life [2]. In occupational therapy (OT), "occupations" refer to the everyday activities individuals engage in, both alone and in the social context, to occupy time and bring meaning and purpose to their lives [2].

Since many of the impairments that harm the health of cancer survivors are amenable to OT [3], OT should be provided to survivors regardless of the type of cancer. The goals in OT are developed collaboratively with the cancer survivor to identify the activities most important to their QOL [3]. Goal-setting is a formal process in which a rehabilitation professional or team, together with the patient and/or their family, negotiates and establishes goals [4]. Successful OT goal-setting is believed to contribute to improving QOL by promoting participation in meaningful occupations. In fact, a systematic review of community-based OT programs for cancer survivors revealed that programs involving collaboration between cancer survivors and occupational therapists were effective in improving motivation, activities of daily living, and QOL [5]. However, OT remains underutilized among cancer survivors [3]. Additionally, setting specific and attainable goals is particularly challenging when cancer survivors exhibit low motivation [6].

These findings underscore the need for programs that incorporate strategies to prevent failures in goal-setting, which are expected to be developed in the future. We identified two effective elements of OT for setting realistic and motivating goals. Element A includes achievement-related goals (e.g., gaining prosperity, improving material conditions, career development, and achieving social prestige), as well as health-related, social, leisure, and psychological goals [7]. By planning goals across multiple categories, cancer survivors can experience partial success in some objectives even when complete achievement is not feasible [8]. Therefore, Element A emphasizes the inclusion of multiple goal categories from these five domains.

In general, there are two types of goals: learning goals and performance goals [9]. Learning goals focus on strategies, processes, and procedures to master tasks (e.g., "learn how to increase the steps"). In contrast, performance goals are set to achieve specific tasks according to certain proficiency criteria (e.g., "carry out moderate aerobic exercise"). However, performance goals may hinder individual progress if anxiety or pressure is high or if achieving the goal seems unlikely. As a result, learning goals are recommended under these circumstances [9]. Element B involves setting learning goals to foster success.

Here, we present the case of a cancer survivor whose occupational therapist incorporated learning goals across the two categories.

## Case presentation

The participant was a 73-year-old man diagnosed with diffuse large B-cell lymphoma and brain metastases. Three years before OT, he was diagnosed with cancer and received chemotherapy and radiation therapy, including whole-brain irradiation. Then, he experienced a frozen gait, short-stepped gait, and slow movements. Post-radiation Parkinsonism, caused by radiotherapy of the basal ganglia, was suspected [10].

One year after cancer treatment, he retired from his job and his wife died, resulting in loneliness. The participant began attending a senior daycare center to facilitate outings and receive assistance with bathing. Additionally, he began receiving home-visit physical therapy twice weekly.

Two years after cancer treatment, he attended four sessions of our once-a-week, one-hour OT program at a daycare center because his ability to perform daily activities had not improved despite ongoing physical therapy. He readily agreed to participate in OT, stating "Even if I cannot fully return to my life before being diagnosed with cancer, I want to maintain my current condition." At OT initiation, he was able to eat and use the toilet independently, though slowly, due to reduced movement. He spent the entire day at home on his bed watching TV.

Evaluation findings and goal-setting

The Functional Assessment of Cancer Therapy-General (FACT-G) [11] was used to assess the participant's QOL, and the Canadian Occupational Performance Measure (COPM) [12] was used to set goals (Table 1). During the initial session, he was less motivated due to activity limitations and did not desire to perform activities other than walking; therefore, goal-setting was postponed. Even if only health-related goals from Element A were set, they would not have been achieved due to a lack of improvement in his gait over the past two years, despite participating in physical therapy. Furthermore, if walking-related performance goals were set based solely on his narrative during the first session, his emotional well-being would have declined further because the likelihood of achieving those goals was low. Additionally, he would have been highly anxious, as his score for "I am losing hope in the fight against my illness" on the FACT-G was low. Therefore, the COPM was used again to set learning goals (Element B), rather than health-related performance goals, during the second session. He agreed to this intervention, setting the learning goal of "knowing how to increase time for enjoyment" (Table 2). No specific activities were set in addition to this learning goal.

**Table 1:** FACT-G and COPM scores of the participant Pre-intervention FACT-G scores were assessed at the first session, and COPM scores were assessed at the second session. Post-intervention FACT-G and COPM scores were assessed at the fourth session. *: The improvement in FACT-G indicated values equal to or above the minimum significant difference. FACT-G: Functional Assessment of Cancer Therapy-General; COPM: Canadian Occupational Performance Measure

	Pre	Post
FACT-G		
Total	59.5	69.6*
Physical well-being	14.0	17.0*
Social/family well-being	17.5	18.6
Emotional well-being	10.0	14.0*
Functional well-being	18.0	20.0*
COPM		
Performance score	3.0	5.0
Satisfaction score	1.0	4.0

**Table 2:** Summary of occupational therapy sessions FACT-G: Functional Assessment of Cancer Therapy-General; COPM: Canadian Occupational Performance Measure

Session number	Evaluation or intervention contents
Session 1	Initial evaluation (FACT-G, COPM)
	Postponement of goal-setting
Session 2	Initial evaluation (COPM)
	Goal-setting of "knowing how to increase time for enjoyment"
	Program: playing Gobang (individual session)
Session 3	Program: playing Gobang (group session)
Session 4	Program: playing Gobang (group session)
	Final evaluation (FACT-G, COPM)

Intervention programs and their process

During the first week after starting the intervention, the participant did not engage in any leisure activities. The intervention program for sessions 2 through 4 was designed to create a pleasant experience and reduce loneliness through Gobang (a game that could be played with others), a pastime from the participant's youth. In the second session, the participant and occupational therapist played Gobang (addressing leisure and psychological goals in Element A) (Table 2). As a result, the leisure domain in Element A was classified as enjoying Gobang, and the psychological domain was classified as reducing loneliness. This marked the first instance of incorporating multiple categories within Element A.

In the third and fourth sessions, the participant played with other clients and daycare staff (addressing social goals in Element A) to ensure the continuation of Gobang after OT. Therefore, the social domain in Element A was classified as interacting with others. The participant looked forward to the weekly Gobang sessions. Surprisingly, by the fourth session, he became motivated to engage in activities of his own choice and began spontaneously going out to karaoke and dining out with friends, with the support of his family.

Outcomes

The results of re-evaluation using the FACT-G and COPM are shown in Table 1. The improvement in the FACT-G indicated values equal to or above the minimum significant difference (total well-being, 3-7; physical well-being, 2-3; emotional well-being, 2; and functional well-being, 2-3) [13]. COPM performance and satisfaction scores improved; however, they did not exceed the minimum significant difference for community-dwelling older individuals (performance score, 3.0; satisfaction score, 3.2) [14].

## Discussion

In the present case, the cancer survivor receiving OT was unmotivated to set goals and did not consider activities other than walking during the initial session. At the second session, an OT program incorporating Elements A and B was introduced, and a learning goal, which included multiple goal categories, was set. Following this, the participant began going out to karaoke and dining out, leading to an improvement in his QOL. This improvement may be attributed to the fact that even when faced with unattainable goals, the occupational therapist could recall non-health-related goals from Element A. Additionally, the occupational therapist's awareness that participation in Gobang would positively impact the participant's health, psychological, and social well-being significantly influenced these results. Therefore, it is important to emphasize the role of the occupational therapist in this intervention, and further research is needed to explore the impact of this program when implemented by different professionals.

Moreover, learning goals set in Element B are effective for complex tasks [15] and have been significantly correlated with metacognition, which involves the ability to plan and control one's own learning process [16]. This intervention may have stimulated the participant's metacognitive abilities, which in turn led to behaviors such as attending karaoke and dining out. Thus, it is possible that this intervention facilitated goal-setting by providing alternative options for the participant, ultimately improving his QOL. This is supported by evidence that active client involvement in goal-setting contributes to better psychosocial outcomes, including QOL and emotional well-being [17].

This case report is unique due to the limited evidence regarding the use of learning goals (Element B) for physical activity [9] and to the fact that, to our knowledge, no OT program has been reported that includes a combination of Elements A and B preventing goal-setting failure. However, as this case report was evaluated only before and after the OT program, the effects during the follow-up period were not investigated. Furthermore, as we included only one participant with diffuse large B-cell lymphoma and brain metastases, further research is needed to determine whether this OT program is effective for other cancer survivors with different types of cancer. Since the success or failure of goal-setting is not exclusive to diffuse large B-cell lymphoma or brain metastases, future research should clarify the long-term effects of including participants with a range of cancer types. Specifically, investigating the impact of this program on the success rate of goal-setting and QOL is warranted.

## Conclusions

The participant in this OT program was initially focused on improving his walking, which caused goal-setting to be delayed. Our case report demonstrated that the program, which includes two key elements (planning goals across multiple categories and setting learning goals), can prevent goal-setting failure. Furthermore, the participant became motivated, and his QOL, particularly his emotional well-being, improved. Future research is needed to investigate the impact of our OT program on the success rate of goal-setting and participants' QOL when this program is structured.
